# Occurrence of the invasion associated marker (*iam*) in *Campylobacter jejuni *isolated from cattle

**DOI:** 10.1186/1756-0500-4-570

**Published:** 2011-12-30

**Authors:** Yasser M Sanad, Issmat I Kassem, Zhe Liu, Jun Lin, Jeffrey T LeJeune, Gireesh Rajashekara

**Affiliations:** 1 Food Animal Health Research Program, Ohio Agricultural Research and Development Center, Department of Veterinary Preventive Medicine, The Ohio State University, Wooster, OH 44691, USA; 2 Department of Animal Science, The University of Tennessee, Knoxville, TN 37996, USA

**Keywords:** *Campylobacter jejuni*, Cattle, Invasion, Host colonization, Invasion associated marker (*iam*)

## Abstract

**Background:**

The invasion associated marker (*iam*) has been detected in the majority of invasive *Campylobacter jejuni *retrieved from humans. Furthermore, the detection of *iam *in *C. jejuni *isolated from two important hosts, humans and chickens, suggested a role for this marker in *C. jejuni*'s colonization of multiple hosts. However, no data exist regarding the occurrence of this marker in *C. jejuni *isolated from non-poultry food-animals such as cattle, an increasingly important source for human infections. Since little is known about the genetics associated with *C. jejuni*'s capability for colonizing physiologically disparate hosts, we investigated the occurrence of the *iam *in *C. jejuni *isolated from cattle and assessed the potential of *iam*-containing cattle and human isolates for chicken colonization and human cell invasion.

**Results:**

Simultaneous RAPD typing and *iam*-specific PCR analysis of 129 *C. jejuni *isolated from 1171 cattle fecal samples showed that 8 (6.2%) of the isolates were *iam*-positive, while 7 (54%) of human-associated isolates were *iam*-positive. The *iam *sequences were mostly heterogeneous and occurred in diverse genetic backgrounds. All *iam*-positive isolates were motile and possessed important genes (*cad*F, *cia*B, *cdt*B) associated with adhesion and virulence. Although certain *iam*-containing isolates invaded and survived in INT-407 cells in high numbers and successfully colonized live chickens, there was no clear association between the occurrence, allelic sequence, and expression levels of the *iam *and the aforementioned phenotypes.

**Conclusions:**

We show that the prevalence of *iam *in cattle *C. jejuni *is relatively lower as compared to isolates occurring in humans and chickens. In addition, *iam *was polymorphic and certain alleles occur in cattle isolates that were capable of colonizing and invading chickens and human intestinal cells, respectively. However, the *iam *did not appear to contribute to the cattle-associated *C. jejuni*'s potential for invasion and intracellular survival in human intestinal cells as well as chicken colonization.

## Background

*Campylobacter jejuni *is an important foodborne pathogen that can cause a variety of infections in humans [1]. Additionally, *C. jejuni *colonizes important food-animals such as chicken and cattle, which together constitute an important source for human infections with this pathogen [2]. Although *C. jejuni *can occur in multiple hosts, it is more readily transmissible within species [3]. Once established in a new host, *C. jejuni *has a remarkable capacity for acquiring genetic material that facilitates adaptation to the host environment [3]. Efficient host colonization can be essential in pathogenesis mechanisms, including host cell invasion and associated sequelae [4]. Furthermore, numerous studies, using live chicken models and in vitro human cell lines, have suggested multiple genetic determinates that are important in *C. jejuni*'s host colonization [4,5]. However, little is known about genetic factors that might be important for *C. jejuni*'s adaptation to multiple hosts, which is important since the broad host range of *C. jejuni *complicates on-farm control measures aimed at decreasing its transmission to humans.

An important factor in *C. jejuni*'s host colonization is its capability to attach to- and/or invade epithelial cells in the host's gastrointestinal tract [6]. Yet, different strains of *C. jejuni *display varying capacities for cellular adherence and invasion, which could be attributed to the presence, absence and/or acquisition of certain genetic determinants that contribute to the pathobiology of this bacterium [7,8]. Although invasion and host adaptation are influenced by the interaction of multiple genetic factors, several individual components, including outer-membrane proteins and secreted antigens can impact *C. jejuni*'s adherence to and invasion of enterocytes [7,9,10]. Of particular interest is the invasion associated marker (*iam*) that was significantly associated with invasive *Campylobacter *[11]. This marker was discovered using random amplified polymorphic DNA (RAPD) analysis that identified a diagnostic DNA band (1.6 Kb) containing a genetic element (designated later as *iam*). Although specific PCR analysis showed that the *iam *was observed in 63% of the invasive isolates retrieved from diarrheic children, the marker was not detected in every potentially invasive isolate and occured in a low percentage of the non-invasive ones. Consequently, it was concluded that mutations/allele variations might have impacted both the detection of the marker and its role in mediating the invasion [11]. However, these assumptions were not tested further and the role of *iam *in *C. jejuni*'s invasion and adaptation to hosts, whether humans or animals, has not been fully investigated. The limited data available suggest that the occurrence of the *iam in C. jejuni *might be both dependent on the characteristics of the human population understudy and associated sources of infection [12,13]. The latter is important since the majority of *Campylobacter *isolated from chicken carcasses, an established source for *Campylobacter *infections, also possessed the *iam *[13,14], which suggests that the *iam *might play a role in the transmission of *Campylobacter *and/or its adaptation to different host(s). Since no data are available concerning the occurrence of *iam *in *C. jejuni *isolated from other hosts, the aforementioned conclusion regarding the *iam *association with multiple-host colonization needs further analysis. Consequently, it was important to investigate the occurrence of *iam *in *C. jejuni *from other important sources such as cattle [15-17] and test their potential for colonization of humans and chickens, respectively. If *iam *is associated with *C. jejuni*'s potential for colonization of multiple hosts, this would facilitate understanding the interactive impact of major animal sources such as chicken and cattle in the transmission of *Campylobacter*. Therefore, we investigated the occurrence of the *iam *in *C. jejuni *isolated from cattle (n = 1171) and determined the association of this element and its alleles with the pathogen's invasion potential of a human intestinal cell line and colonization of 1-day old chickens.

## Methods

### Isolation of *Campylobacter jejuni *from cattle and human samples

Fecal samples (n = 1171) were collected from cattle at 4 geographic locations (North, Mid-West, East, South) across the U.S. To isolate *C. jejuni*, 1 g of each fecal sample was enriched in Preston broth for 48 h at 42°C under microaerobic conditions (5% O_2_, 10% CO_2_, and 85% N_2_). From the enrichments that showed growth, an inoculum (100 μl) was spread onto modified Cefoperazone Charcoal Deoxycholate Agar (mCCDA) plates, which were then incubated for an additional 48 h at 42°C under microaerobic conditions [18]. Colonies exhibiting typical *Campylobacter *phenotype (flat and grey with metallic sheen) were selected from the plates and subjected to species-specific PCR analysis to confirm their identity [19,20]. The size of the PCR products was determined using a 1 Kb DNA ladder and detection was confirmed by comparison to a PCR product generated from a *C. jejuni *81-176 (wild-type strain), which was used as a positive control in all PCR analysis. Negative controls (reactions with no DNA templates) were included in all PCR analysis to ensure specific product amplification.

Additional *C. jejuni *isolates from human hosts were acquired from a medical center (The Ohio State University). These isolates represented different sporadic human infections and their identity was further confirmed using the aforementioned PCR analysis.

### Detection of *iam *using RAPD typing and PCR

To determine if the *C. jejuni *originating from cattle and human samples carried the *iam *locus (a diagnostic 1.6 Kb band), all isolates were subjected to DNA fingerprinting using RAPD analysis as described in Carvalho et al. [11]. RAPD-PCR products were then analyzed using 1.4% agarose gels, containing 0.5 μg/ml of ethidium bromide. RAPD fingerprints were documented and dendrograms were constructed using BioNumerics 5.1 software (Applied Maths, Inc, USA).

*C. jejuni *isolates that carried the *iam *locus as identified by the RAPD fingerprinting were tested to further confirm the presence of the *iam *locus. This was achieved using PCR analysis to detect a 518-bp DNA fragment inside the 1.6 kb band that was earlier identified as the *iam *locus by RAPD fingerprinting as described in Carvalho et al. [11].

### Detection of virulence-associated genes using PCR

*C. jejuni *isolates that were identified as *iam*-positive using both RAPD and *iam*-specific PCR analysis, hereafter referred to as the *iam*-positive *C. jejuni*, were screened for genes that are important in the pathobiology of this pathogen. Specifically, PCR analysis was performed for the detection of the *cad*F (*Campylobacter *adherence factor), *cia*B (*Campylobacter *invasion antigens), and *cdt*B (cytolethal distending toxin) genes as described elsewhere [10,21,22]. Genomic DNA from *C. jejuni *strain 81-176 was used as a positive control, while negative controls contained the PCR reagent mix with no DNA templates.

### Motility assay

To establish that *iam*-positive *C. jejuni *were putatively capable of host invasion and colonization, it was important to establish that the isolates were not defective in motility. For this purpose, the motility of the *iam*-positive *C. jejuni *was tested using semi-solid (0.4%) Mueller-Hinton (MH) agar plates as described previously [23]. The diameter of the zone of motility was measured and compared to that of *C. jejuni *81*-*176 (positive control). The motility assays were repeated twice for each isolate, which were also tested in duplicates per each assay.

### In vitro cell invasion and intracellular survival assay using human epithelial cell lines (INT-407)

The human intestinal cell invasion assays were performed using *iam*-negative and *iam*-positive *C. jejuni *isolated from both cattle and humans. For this purpose, 10^5 ^cells ml^-1 ^of INT-407 (human embryonic intestine, ATCC CCL 6) were seeded into each well of a 24-well tissue culture plates in Minimum Essential Medium Eagle (MEM, Fisher scientific, USA) supplemented with 10% fetal bovine serum (FBS, Fisher scientific, PA, USA). The plates were then incubated at 37°C in a humidified incubator with 5% CO_2 _until semi-confluent mono-layers were obtained [24,25]. For infection with *C. jejuni*, the INT-407 mono-layers were washed three times and covered in MEM supplemented with 1% FBS. Similarly, the *C. jejuni *cultures were washed three times and suspended in MEM supplemented with 1% FBS to obtain 10^7 ^bacteria ml^-1^. One ml of bacterial suspension was added to each well containing the INT-407 semi-confluent monolayer, achieving a 1:100 multiplicity of infection (MOI). After 3 h of incubation, cells were treated with gentamicin (150 μg ml^-1^) for 2 h to inhibit the bacteria that did not invade the cells. The infected mono-layers were washed with 1× PBS, lysed using 0.01% Triton X-100 and serially diluted (10-fold) in 1× PBS. One hundred μl of each dilution were spread on MH agar plates. The agar plates were then incubated for 48 h at 42°C under microaerobic conditions, after which colony forming units (CFU) were counted. Each isolate was tested in duplicate per assay, while the experiment was repeated twice on separate occasions. *C. jejuni *81-176 and NCTC11168 were used as controls in all invasion assays. A negative control that consisted of a well containing only INT-407 with no bacteria was processed in parallel to the infected monolayers.

For the intracellular survival assays [26], *Campylobacter *cultures and the INT-407 cells were processed as described above. However, after treatment with gentamicin, the monolayers were covered with MEM containing 1% FBS and gentamicin (10 μg ml^-1^) and incubated for additional 24 h at 37°C. Then the monolayers were washed three times with MEM containing 1% FBS, lysed and processed as described above. The number of viable intracellular bacteria was determined by counting CFUs.

### Phylogenetic analysis of the *iam *alleles

To determine if the *iam *sequences were heterogeneous and examine relationships between the *iam *occurring in cattle *C. jejuni *isolates and those from human samples, the 518 bp *iam *fragments were sequenced and subjected to a phylogenetic analysis. Briefly, *iam*-specific PCR products were purified using the QIAquick PCR purification kit (Qiagen, CA, USA) and commercially sequenced (Molecular and Cellular Imaging Center, OARDC, OH, USA). The identity of the sequences was confirmed by BLAST analysis. The sequences were then exported to MEGA4 software [27], aligned and analyzed. The phylogenetic tree was drawn using the Neighbor-Joining method to determine the evolutionary relationship among the sequences. The *iam *sequences that were analyzed in this work were deposited in GenBank under accession numbers: HM533957-HM533968, JF927289-JF927291 and HQ317917.

### Expression analysis for the *iam *using quantitative real-time PCR (q-RT PCR)

Quantitative reverse transcriptase (RT)-PCR was used to investigate an association between the expression of *iam *and the phenotypes of the cattle-associated *C. jejuni*. The q-RT PCR primers (Table 1) targeted *iam *conserved sequences, which were determined by multiple alignment of the *iam *alleles using ClustalW2 (http://www.ebi.ac.uk/Tools/msa/clustalw2/). In order to ensure the detection of expression of different alleles, two sets of q-RT PCR primers were designed to target different fragments of the *iam *using Beacon Designer 7.0 software (Premier Biosoft International, Palo Alto, CA). A third set of q-RT PCR primers (Table 1) was specifically constructed to target the *iam *of the hyper-invasive *C. jejuni *81-176. Total RNA was extracted (RNeasy Mini kit, Qiagen) from the *iam*-containing strains and two *iam*-negative strains, which were included as controls. Subsequently, cDNA was synthesized (SuperScript III First-Strand Synthesis SuperMix, Invitrogen) and used for q-RT PCR (SensiMixPlus SYBR RT-PCR kit, Quantance) in a Mastercycler ep realplex^2 ^thermal cycler (Eppendorf). The q-RT PCR analysis was repeated three times with two replicates in each assay and threshold cycle (*C_T_*) values for each sample were then averaged to represent the expression levels of the *iam*.

**Table 1:** List of primers used in this study

Primer	Sequence (5 '-3 ')	Properties	Product size	**Ref**.
16S rRNA-F 16S rRNA-R	5'-ATCTAATGGCTTAACCATTAAAC-3 '5 '-GGACGGTAACTAGTTTAGTATT-3 '	*Campylobacter *specific	850 bp	[19]

*map*A-F *map*A-R	5 '-CTATTTTATTTTTGAGTGCTTGTG-3 ' 5 '-GCTTTATTTGCCATTTGTTTTATTA-3 '	*C. jejuni *specific	589 bp	[20]

*cad*F-F *cad*F-R	5 '-TTGAAGGTAATTTAGATATG-3 ' 5 '-CTAATACCTAAAGTTGAAAC-3 '	*cad*F detection	400 bp	[10]

*cdt*B-F *cdt*B-R	5 '-GTTAAAATCCCCTGCTATCAACCA-3 ' 5 '-GTTGGCACTTGGAATTTGCAAGGC-3 '	*cdt*B detection	495 bp	[22]

*cia*B-F *cia*B-R	5 '-TTTTTATCAGTCCTTA-3 ' 5 '-TTTCGGTATCATTAGC-3 '	*cia*B detection	986 bp	[21]

Primer 1290	5 '-GTGGATGCGA-3 '	RAPD-typing	Variable	[11]

1.6-F 1.6-R	5 '-GCGCAAAATATTATCACCC-3 ' 5 '-TTCACGACTACTATGCGG-3 '	*iam*-specific	518 bp	[11]

Iam1-F Iam1-R	5 '-AACATTAGCGAGGAAGAT-3 ' 5 '-GTATATTCTTTAAGAGGGGTAG-3 '	*iam*-specific (qRT-PCR)	160 bp	This study

Iam2-F Iam2-R	5 '-AACATTAGCGAGGAAGAT-3 ' 5 '-TCATTTAAACCGACCATTT-3 '	*iam*-specific (qRT-PCR)	160 bp	This study

Iam- 81176-F Iam- 81176-R	5 '-AAGATAGCATACAAGAACT-3 ' 5 '-ATTCACGACTACTATAAGG-3 '	*iam*-81176-specific (qRT-PCR)	160 bp	This study

### Typing of *iam*- positive *C. jejuni *using pulsed field gel electrophoresis (PFGE)

To determine the relatedness and diversity of genomic backgrounds of *iam*-positive *C. jejuni*, these isolates from cattle and human samples were analyzed using PFGE as described by Ribot et al. [28]. The resulting PFGE patterns were documented and analyzed using the BioNumerics 5.1 software (Applied Maths Inc, TX, USA). Similarity and clustering analysis of the PFGE patterns were performed using the Dice Coefficient and the unweighted pair-group method with arithmetic averages, UPGMA (optimization of 1% and position tolerance of 1.5%), respectively.

### In vivo chicken colonization assay

**Figure 1:**
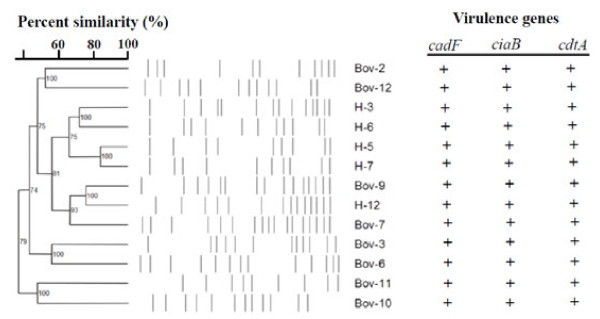
**Dendrogram showing the pulsed-field gel electrophoresis profile for the *iam*-positive *C. jejuni***. H-9 and H-13 could not be typed using PFGE. Numbers on the nodes represent the Cophenetic correlations. Virulence genes detected by PCR in *iam*-positive *C. jejuni *were included for adjacent to the corresponding PFGE profile.

*iam*-positive *C. jejuni *were selected for in vivo chicken colonization assays based on *iam*-sequence type and the *iam *expression profile. Subsequently, Bov-6 was selected to represent Bov-9, Bov-10, and 11 (Figure 1), while Bov-9 was excluded because it did not express the *iam *product. One day-old chicken (specific pathogen free) were divided into groups, each containing seven birds. Before the experimental infection, the chickens were confirmed to be *Campylobacter *free by testing cloacal samples collected from each individual. Groups were divided to simultaneously test *iam*-positive isolates versus *iam*-negative ones and isolates from human hosts versus those from cattle. Subsequently, *C. jejuni *isolates were suspended in MH broth to achieve an OD_600 _of 0.04 and individual chickens in each group were inoculated orally with 200 μl of the suspensions (~2 × 10^5 ^CFU), respectively. Seven days post-inoculation, the chickens were euthanized and the caeca were aseptically collected, weighed, and homogenized in 1X PBS (pH 7.4). The caecal extracts were serially diluted (10-fold) and 100 μl from each dilution were spread onto MH agar plates supplemented with SR117E (Oxoid, KS, USA), a *Campylobacter *selective supplement. The plates were then incubated at 42°C under microaerobic conditions for 48 h and the number of CFU/g of caecal contents was calculated to determine the colonization capability of the *C. jejuni *isolates. The chickens were cared for according to the guidelines of the Association for the Assessment and Accreditation of Laboratory Animal Care (AAALAC).

### Statistical analysis

Data were presented as means ± standard error (SE) and assessed using analysis of variance (ANOVA), followed by Tukey's significance test. A *P *value of < 0.05 was used to indicate if differences were statistically significant.

## Results and discussion

A total of 129 *C. jejuni *were isolated from fecal samples (n = 1171) collected from cattle in 4 geographic locations (North, South, Midwest, East) in the U.S. Additionally, 13 *C. jejuni *isolates were acquired from different sporadic human infections. The identity of the *C. jejuni *isolates was confirmed using PCR. RAPD analysis was performed as described by Carvalho et al. [11] and showed that 10.8% of the cattle isolates possessed the 1.6 Kb DNA band that potentially harbors *iam *as compared to 54% of the human isolates. Furthermore, *iam*-specific PCR [11] showed that 6.8% of the cattle isolates were *iam*-positive as compared to 54% of the human isolates. Randomly selected isolates that did not show the 1.6 Kb band after RAPD analysis were also observed to be *iam*-negative using *iam*-specific PCR analysis. The discrepancy between the RAPD and the PCR results for the cattle isolates was not surprising since, as suggested by Carvalho et al. [11], the PCR primers might not necessarily detect *iam *mutated fragments/alleles. Additionally, mutations might spuriously give rise to a 1.6 Kb band that can be mistaken for the *iam *locus. Therefore, in order to limit *iam *false-positives that might be detected using either method, we selected isolates that possessed both the 1.6 Kb fragment and the *iam*-PCR product, referred to as *iam*-positive strains, for further analysis.

**Figure 2:**
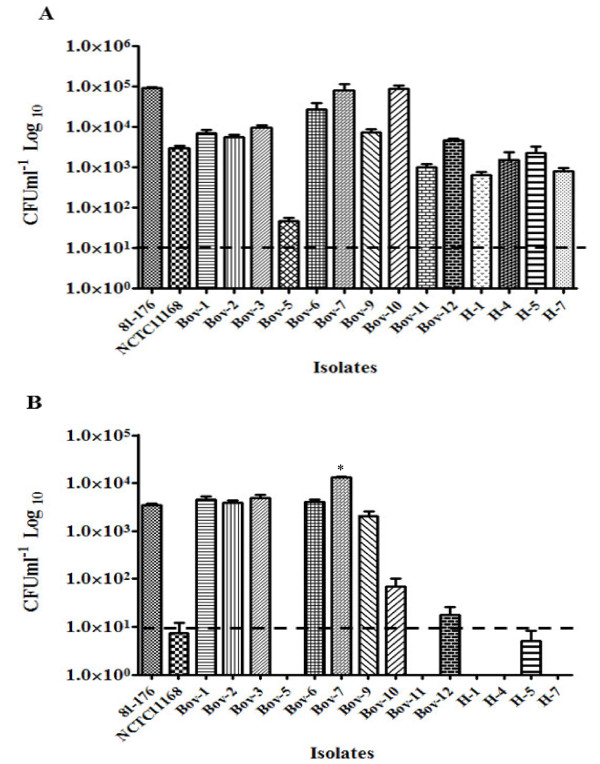
**Interaction of iam-containing C. jejuni with a human intestinal epithelial cells (INT-407). A.*** C. jejuni *invasion potential of human INT-407 cells. **B.** Intracellular survival of *C. jejuni *isolates in INT-407 cells. Bov-1, Bov-5, H-1, and H-4 are *iam*-negative *C. jejuni *used for comparison with *iam*-containing isolates. *C. jejuni *81-176 and NCTC11168 are wildtype strains used as controls. Data were log transformed and presented as mean ± SE (standard error). Statistical significance was determined at *P *< 0.05.

The *iam*-positive cattle isolates and 2 *iam*-positive human isolates (H-5, H-7) were assessed for their capability to invade and survive in human intestinal cells (INT-407) [5,29]. Two *iam*-negative cattle isolates (Bov-1, Bov-5) and 2 *iam*-negative human isolates (H-1, H-4) were used for comparison. Although all the *iam*-positive strains were motile on semi-solid Mueller-Hinton agar (data not shown), our results show that 5 (Bov-2, 3, 6, 7, and 10) of the *iam*-positive cattle isolates invaded INT-407 with numbers higher than those of *C. jejuni *NCTC11168. Additionally, two isolates (Bov-7 and Bov-10) were also more invasive as compared to the *C. jejuni *81-176, a highly invasive strain (Figure 2A). Furthermore, 4 of the *iam*-positive cattle isolates (Bov-2, 3, 6, and 7) exhibited either equivalent or higher capacity for intracellular survival in INT-407 as compared to *C. jejuni *81-176 (Figure 2B). In contrast, independent of the occurrence of the *iam*, all tested human isolates exhibited significantly decreased capacity for invasion and intracellular survival in the INT-407 cell line as compared to *C. jejuni *81-176 (Figure 2A and 2B). Consequently, the occurrence of the *iam *in the cattle and human isolates did not seem to confer any clear advantage in terms of invasiveness and the intracellular survival potential in the human cell line. However, since the *iam *was previously detected in 16% of non-invasive *Campylobacter *[11], Carvalho et al. [11] speculated that the non-invasive strains might carry a mutated variant of this marker and that different *iam *alleles might result in discrepancies in the invasion potential of disparate *C. jejuni *strains. This is plausible since allelic variations in other targets were also suggested to impact the virulence of different strains [7,30].

**Figure 3:**
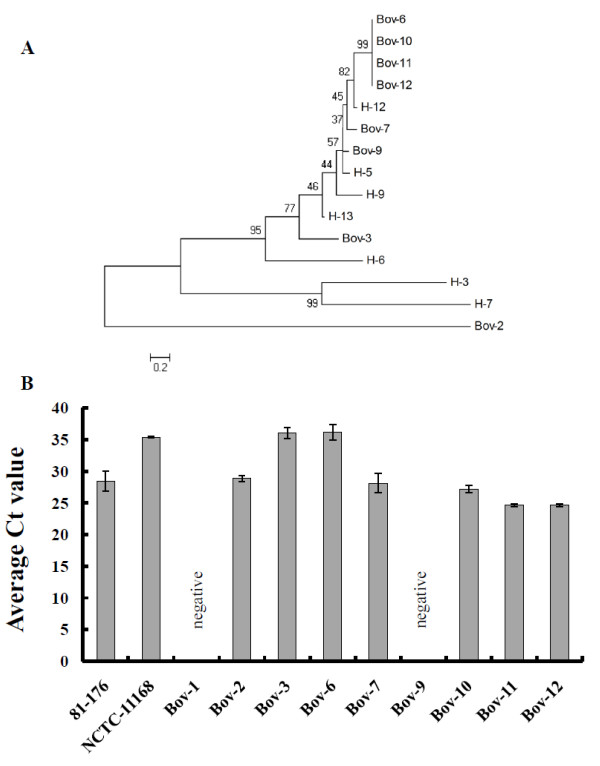
**The evolutionary relationship between iam sequences and the expression profiles of the iam locus in cattle- and human isolates. A.** The evolutionary relationship between *iam *sequences detected in *C. jejuni *isolated from human and cattle samples. The tree was drawn to scale using the Neighbor-Joining method. The percentage of replicate trees in which the *iam *sequences clustered together in the bootstrap test (10000 replicates) is shown next to the branches. The evolutionary distances are in the units of the number of base substitutions per site. **B**. Expression of *iam *as determined using qRT-PCR analysis. Bov-1 is an *iam*-negative strain. Wildtype strains (*C. jejuni *81-176 and NCTC11168) that harbor the *iam *were also included for comparison with *iam*-containing cattle isolates. The data represented was collected using the primer set iam1; however, set iam2 resulted in similar results (data not shown). Expression in *iam*-negative strains was not detectable even after 35 cycles of amplification.

Genomic analysis of the *iam *sequence deposited by Carvalho et al. [11] showed that the *iam *encodes an ABC transporter (Table 2). This is important since potential virulence factors, including surface structures such as transporters, are predicted to harbor much of the genetic diversity that characterize disparate *C. jejuni *strains [3]. Interestingly, the *iam *was only 84% and 83% similar to sequences harbored in *C. jejuni *11168 and 81-176, respectively (Table 2) and 86% similar to sequences [Genbank:HQ317917] that occurred in a newly sequenced cattle isolate; *C. jejuni *JL11 [31], which highlights the polymorphism of this marker. To further examine the extent of sequence polymorphism in the *iam *occurring in cattle *C. jejuni *isolates, we sequenced the 518 bp *iam *fragment. Subsequent phylogenetic analysis showed that only 4 cattle isolates (Bov-6, 10, 11, and 12) shared the same *iam *sequence type, while the other *iam *sequences from cattle and humans were heterogeneous, clustering in groups that mostly contained *iam*-isolates from both hosts (Figure 3A). Interestingly, the cattle isolates with identical *iam *sequences (Bov-6, 10, 11 and 12), did not exhibit similar invasion and intracellular survival properties in INT-407 cells (Figure 2A and 2B), which suggested that there were no clear associations between an *iam *sequence type and the aforementioned phenotypes. Furthermore, the *iam *also did not appear to affect the invasion and intracellular survival potential in the tested human strains, as both *iam*-positive and *iam*-negative isolates showed similar properties, respectively.

**Table 2:** Analysis of the *iam *marker using the BLAST algorithm.

Description	Accession	Locus Tag	Query coverage (%)	Similarity (%)
*C. jejuni *hypothetical integral membrane protein (iamB) gene (partial cds); and ABC transporter (iamA) gene (complete cds)	AF023133.1	AF023133	100%	100%

*C. jejuni *subsp. *jejuni *IA3902	CP001876.1	CJSA_1559	100%	84%

*C. jejuni *subsp. *jejuni *NCTC 11168	AL111168.1	Cj1647	100%	84%

*C. jejuni *RM1221	CP000025.1	CJE1819	100%	84%

*C. jejuni *subsp. *jejuni *81116	CP000814.1	C8J_1549	100%	83%

*C. jejuni *subsp. *jejuni *81-176	CP000538.1	CJJ81176_1638	100%	83%

*C. jejuni *subsp. *jejuni *JL110034*	NA	NA	100%	86%

*C. jejuni *subsp. *doylei *269.97	CP000768.1	JJD26997_2007	100%	85%

*C. lari *RM2100	CP000932.1	Cla_0118	94%	81%

The sequence reported by Carvalho et al. [11] was used for query. Cds stands for coding sequence. *JL11 is a cattle isolate that was sequenced in Dr. Lin's laboratory; its sequence and complete annotations have not been submitted to Genbank, yet [31]. However, the *iam *homolog in JL11 was deposited in Genbank [Genbank: HQ317917]. NA: Not available

It is possible that the presence of *iam *gene sequences might not be necessarily associated with the expression of its products, which might explain the lack of an apparent relationship between *iam *and invasiveness. Therefore, the expression of the *iam *was assayed for the cattle isolates using q-RT PCR, which showed that the expression levels of the *iam *varied between the strains (Figure 3B). Bov-3 and Bov-6 with low *iam *expression levels and Bov-9 with no detectable expression were still capable of invading and surviving in INT-407 cells (Figure 2A and 2B). Although isolates (Bov-7 and Bov-10) with *iam *expression similar to that of *C. jejuni *81-176 exhibited high invasion and intracellular survival potential, isolates with relatively the highest *iam *expression (Bov-11 and 12) did not possess the highest capacities for the aforementioned phenotypes. Since *iam *expression properties in the tested isolates were consistent using two sets of q-RT PCR primers (data not shown), it was concluded that the expression of *iam *did not seem to confer any clear advantage in terms of invasion and intracellular survival.

The virulence traits of *C. jejuni *might likely be affected by the interaction of several genetic elements [9,10,30]. Hence, the role of the *iam *in the pathobiology of *C. jejuni*, if any, would likely depend on other factors (e.g. flagella, adhesins), which in turn might need to occur in specific allelic sequences to mediate their impact. It was interesting to note that the pulsed field gel electrophoresis analysis showed that the genotypes of the *iam*-positive strains were mostly diverse (Figure 1). This indicated that the *iam *is occurring in diverse genotypic backgrounds that, along with the *iam *sequence heterogeneity, might impact the role of this locus in the pathobiology of *C. jejuni*. Subsequently, it was important to investigate whether the *iam*-containing isolates harbored genes that are commonly associated with *C. jejuni *adherence and virulence in order to ensure that our observations can be attributed to *iam *and not other genetic defects. Subsequently, PCR analysis showed that the *iam*-containing isolates carried the *cad*F, *cia*B, and *cdt*A genes (Figure 1) that are important for *C. jejuni *pathogenesis [9,10,12]. Since many of the tested isolates were not defective in invasion of INT-407 cells, PCR detection of the aforementioned virulence genes was satisfactory to further confirm that the *iam*-containing isolates did not appear to be deficient in genes that might be important for attachment and invasion of INT-407 cells.

**Figure 4:**
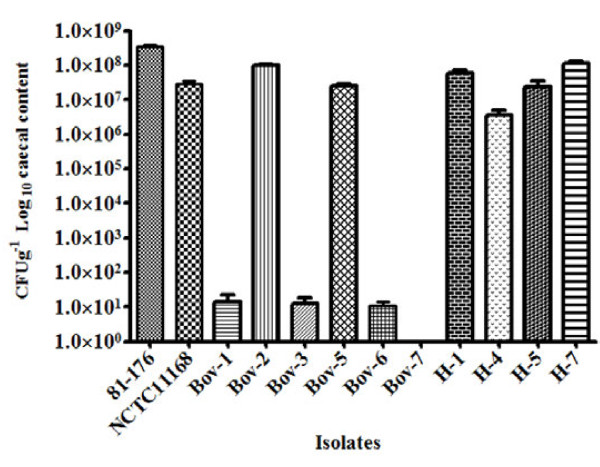
**Chicken colonization with *C. jejuni *isolated from cattle and human hosts**. Results were represented as means of CFU g^-1 ^of caecum retrieved from at least six chicks per isolate tested. Bov-1, Bov-5, H-1, and H-4 are *iam*-negative *C. jejuni *used for comparison with *iam*-containing isolates. *C. jejuni *81-176 and NCTC11168 were used as controls. Data were log transformed and presented as mean ± SE (standard error).

We examined if there was a potential impact for the *iam *in *C. jejuni*'s colonization of chickens. Out of 5 *iam*-containing cattle isolates tested, only one (Bov-2) colonized the chickens with numbers that were significantly higher than those of the *iam*-negative strains (Figure 4). Bov-7 that exhibited relatively high expression of the *iam *was not detected in the chickens (Figure 4). Selected human isolates colonized the chickens in similar numbers, regardless of the presence or absence of *iam*. Therefore, the occurrence and expression of *iam *apparently did not contribute to the chicken colonization potential of *C. jejuni*.

## Conclusions

Carvalho et al. [11] only correlated the occurrence of the *iam *in a certain number of invasive *C. jejuni *using typing techniques, while acknowledging the existence of the marker in a relatively smaller percentage of non-invasive strains. Our analysis was more rigorous and included attempts to associate the *iam *with several important phenotypes. We report that the *iam *does not necessarily contribute to invasion and survival in human intestinal cells or colonization of chickens. Subsequently, the use of the *iam *as a virulence determinant in epidemiological studies (e.g. references 11-14) might be potentially misleading and might require reevaluation. However, it must be noted that despite our extensive sampling efforts, only a limited number of *iam*-containing *C. jejuni *were isolated in this study. This small number of isolates tested in this study necessitates a cautious interpretation of the data regarding the contribution of *iam *to invasive properties of *C. jejuni*. This can be clarified using alternative approaches such as testing deletion mutants of this marker in future experiments.

## Competing interests

The authors declare that they have no competing interests.

## Authors' contributions

Conceived and designed the experiments: IIK YMS GR. Performed and analyzed the experiments: YMS IIK ZL GR. Wrote the paper: IIK, YMS, GR. Reviewed, edited and approved manuscript: IIK YMS GR JL JTL. All the authors read and approved the manuscript.
